# 1028. Real-World Use of Long-Acting Cabotegravir + Rilpivirine in People with HIV with Detectable Viral Loads at Initiation: Findings from the OPERA^®^ Cohort

**DOI:** 10.1093/ofid/ofad500.059

**Published:** 2023-11-27

**Authors:** Ricky K Hsu, Michael Sension, Jennifer S Fusco, Laurence Brunet, Quateka Cochran, Gayathri Sridhar, Vani Vannappagari, Jean A van Wyk, Michael B Wohlfeiler, Gregory P Fusco

**Affiliations:** AIDS Healthcare Foundation\ NYU School of Medicine, New York, New York; CAN Community Health, Fort Lauderdale, Florida; Epividian, Inc., Durham, North Carolina; Epividian, Inc., Durham, North Carolina; Aids Healthcare Foundation, Miami Beach, Florida; ViiV Healthcare, Fairfax, Virginia; ViiV Healthcare, Fairfax, Virginia; ViiV Healthcare, Brentford, UK, Brentford, England, United Kingdom; AIDS Healthcare Foundation, Miami Beach, Florida; Epividian, Inc., Durham, North Carolina

## Abstract

**Background:**

Cabotegravir + rilpivirine (CAB+RPV) injections, the first complete long-acting (LA) antiretroviral therapy (ART) regimen, was approved by the FDA in January 2021 for ART-experienced people with HIV (PWH) who are virologically suppressed (VL < 50 copies/mL). Among individuals virologically non-suppressed (VL ≥ 30 copies/mL) at initiation, high rates of virologic suppression were observed in the demonstration project at Ward 86 for CAB+RPV LA. We observed CAB+RPV LA real-world utilization and effectiveness over the first 2 years of availability in the United States (US) in individuals with VL ≥ 50 copies/mL at initiation in the OPERA® Cohort.

**Methods:**

All ART-experienced adults with VL ≥ 50 copies/mL at initiation who received their first CAB+RPV LA injection between 21Jan2021 and 28Feb2023 were followed until 25Mar2023. Discontinuation (ART switch or 2 consecutive missed doses) and confirmed virologic failure (CVF; 2 consecutive VLs ≥ 200 copies/mL or 1 VL ≥ 200 copies/mL + discontinuation after a VL < 50 copies/mL) were described, for all individuals with VL ≥ 50 copies/mL and for the subset with VL ≥ 200 copies/mL.

**Results:**

Of 1843 ART-experienced PWH with ≥ 1 CAB+RPV injections, 229 (12%) had a VL ≥ 50 copies/mL at initiation. At baseline, they were a median of 41 (interquartile range: 32, 52) years of age, 31% female, 57% Black and 20% Hispanic. Injections started a median of 9 (4, 17) years after HIV diagnosis with a median viral load of 2.1 (1.8, 3.8) log copies/mL and median CD4 count of 579 (350, 759) cells/μL (Table 1). Over a median of 6.1 (3.5, 10.1) months of follow-up, 83% were still on CAB+RPV LA at study end. Among the 80% with ≥ 1 VL over follow-up, 94% had a VL < 200 copies/mL and 75% had a VL < 50 copies/mL at study end. CVF was observed in 7 (4%) individuals (Table 2). Similarly for the subset of 93 individuals with a VL ≥ 200 copies/mL at initiation, 90% and 74% had a VL < 200 or < 50 copies/mL at study end, respectively, when follow-up VLs were available (Table 2).

**Figure:**
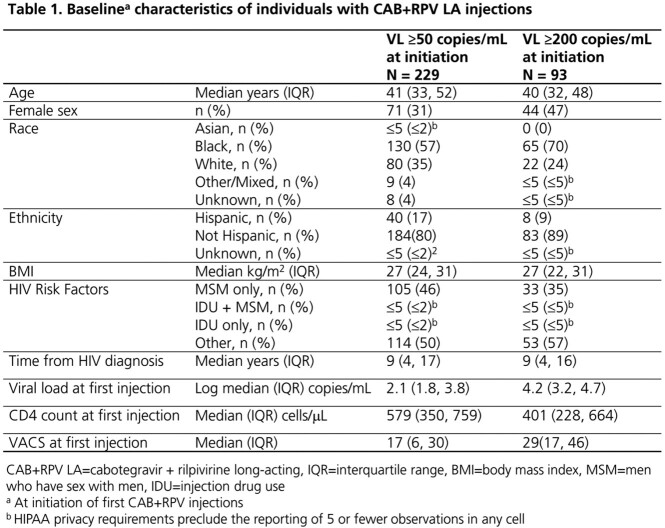

**Figure:**
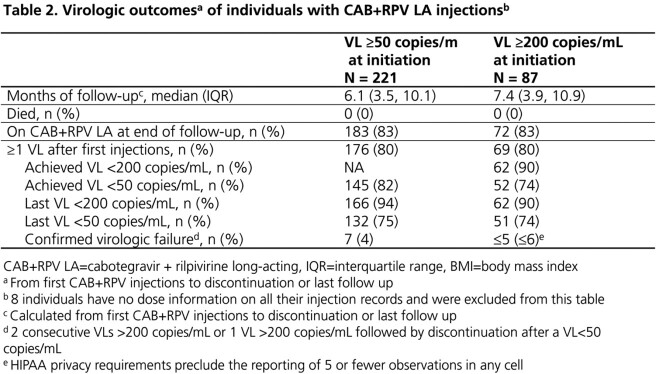

**Conclusion:**

In a large, diverse cohort of real-world, routine clinical care in the US, CAB+RPV LA injections were observed in individuals with viral loads ≥ 50 copies/mL at initiation. Most of these individuals were able to suppress to VL < 50 copies/mL and maintain suppression through study end.

**Disclosures:**

**Ricky K. Hsu, MD**, Gilead Sciences: Advisor/Consultant|Gilead Sciences: Grant/Research Support|Gilead Sciences: Honoraria|Janssen: Advisor/Consultant|Janssen: Grant/Research Support|Janssen: Honoraria|Merck: Advisor/Consultant|Merck: Honoraria|ViiV Healthcare: Advisor/Consultant|ViiV Healthcare: Honoraria **Michael Sension, MD**, Gilead: Advisor/Consultant|Gilead: Honoraria|Viiv: Advisor/Consultant|Viiv: Grant/Research Support|Viiv: Honoraria **Jennifer S. Fusco, BS**, Epividian, Inc.: Salary|Epividian, Inc.: Ownership Interest|Epividian, Inc.: Stocks/Bonds **Laurence Brunet, PhD**, Epividian, Inc.: Salary|Epividian, Inc.: Stocks/Bonds **Gayathri Sridhar, MBBS, MPH, PhD**, GlaxoSmithKline: Stocks/Bonds|ViiV Healthcare: Full Time Employee **Vani Vannappagari, MBBS, MPH, PhD**, GlaxoSmithKline: Stocks/Bonds|ViiV Healthcare: Employee **Jean A. van Wyk, MBChB, MFPM**, ViiV Healthcare Ltd: Stocks/Bonds **Michael B. Wohlfeiler, JD, MD, AAHIVS**, ViiV Healthcare: Serves as a PI on clinical trials, but does not receive personal compensation for this work (it goes directly to AIDS Healthcare Foundation) **Gregory P. Fusco, MD, MPH**, Epividian, Inc.: Board Member|Epividian, Inc.: Ownership Interest|Epividian, Inc.: Stocks/Bonds

